# Application of the One Health Surveillance (OHS) Matrix to Evaluate the Disease Surveillance Systems in Gujarat, India: A Policy Content Analysis

**DOI:** 10.1007/s44197-024-00317-2

**Published:** 2024-10-25

**Authors:** Sandul Yasobant, Ravina Tadvi, Walter Bruchhausen, Deepak B. Saxena

**Affiliations:** 1 Department of Public Health Science, Indian Institute of Public Health Gandhinagar (IIPHG), Opp. New Air Force Station HQ, Nr Lekawada, Gandhinagar, 382042 India; 2 Centre for One Health Education, Research and Development (COHERD), Indian Institute of Public Health Gandhinagar (IIPHG), Opp. New Air Force Station HQ, Nr Lekhawada, Gandhinagar, 382042 India; 3 School of Epidemiology and Public Health, Datta Meghe Institute of Medical Sciences (DMIMS), Wardha, 442107 India; 4 Global Health, Institute for Hygiene and Public Health, University Hospital Bonn, 53127 Bonn, Germany

**Keywords:** OHS, OHSM, One health, Surveillance, Collaboration, India

## Abstract

An effective disease surveillance system can detect outbreaks of emerging zoonotic diseases early and allow the system to respond immediately and control the epidemic. Gujarat is a western Indian state with a population of more than 65 million humans and 26 million livestock, and it has the respective surveillance systems under the Department of Health & Family Welfare for humans and the Department of Animal Husbandry for animals. A veterinarian is placed on establishing cross-sectoral collaborations between these systems, but the joint collaborative activities and their effect on the early warning response are least understood. This provides an opportunity to conduct a secondary policy content analysis study using the One Health Surveillance Matrix (OHSM). The aim of this study was to evaluate the level of collaboration among human-animal disease surveillance systems for zoonotic disease in Gujarat, India. This study findings highlight a few gaps in the human health surveillance system, such as data sharing, sampling, outreach to decision-makers, and external communication, while the animal health surveillance system exhibits deficiencies in the data dissemination to decision-makers, management and storage of data analysis, interpretation of data, and external communication as per the OHSM evaluation. Despite major differences, there is a possibility of developing the One Health Surveillance system in Gujarat, India which is profoundly documented in this study.

**Supplementary Information:**

The online version contains supplementary material available at 10.1007/s44197-024-00317-2.

## Introduction

### One Health Surveillance System, its Need and Practical Advantages

The definition of One Health emphasizes collaboration between various sectors, i.e., those for human, animal, and environmental health, at the local, regional, and national levels to achieve optimal health outcomes. The concept of One Health Surveillance (OHS) describes the systematic collection, validation, analysis, and interpretation of data and the dissemination of information collected on humans, animals, and the environment to inform decisions for more effective and efficient, evidence- and system-based health interventions [1]. Since zoonoses cause 2.5 billion cases of human illness and 2.7 million deaths worldwide each year [2], it is overdue to strengthen the existing surveillance systems that so far lack the implementation of the One Health concept, considering that 75% of infectious diseases are of zoonotic origin [3]. India has a total of 536.76 million livestock population with an increase of over 4.6% compared to the earlier census [4, 5]. In India, the Integrated Disease Surveillance Program– Integrated Health Information Platform (IDSP-IHIP) and National Animal Disease Reporting System (NADRS) collect data on human and animal health status to generate disease alertsThe most important zoonotic diseases reported in India are rabies, brucellosis, toxoplasmosis, cysticercosis, echinococcosis, Japanese Encephalitis (JE), plague, leptospirosis, Scrub typhus, Nipah, trypanosomiasis, Kyasanur forest disease (KFD), and Crimean-Congo haemorrhagic fever (CCHF) [6]. Despite various emerging and re-emerging diseases reported in India, the joint control program for zoonoses is highly limited [7]. Increasing livestock population and human-animal density suggest a need to develop an integrated and multisectoral surveillance system where data collected and analysed by both sectors can be used for early detection and prevention of zoonotic diseases. Advantages are already visible: In two states of India, Odisha and Karnataka, the One Health approach was successfully implemented by a collaboration of ICAR - National Institute of Veterinary Epidemiology and Disease Informatics (ICAR-NIVEDI) for surveillance of Anthrax [5].

### Developing OHS: Issues to the Challenges in LMIC

There are many challenges in developing One Health surveillance in Low- and Middle-income Countries (LMICs), i.e., lack of data sharing, infrastructure, human resources, political commitment, data quality, etc. Collaboration amongst multiple sectors, including the environmental, animal, and human health departments, is necessary for effective One Health surveillance. Due to varying objectives, communication difficulties, and competing resource needs, LMICs frequently struggle to establish interdisciplinary collaboration. One Health Surveillance requires adequate training programs to gather data from the community; the critical issue to address this problem is a lack of policy consistency, constrained financial resources, subpar administration, insufficient leadership, obstacles to data sharing, lack of funding opportunities, and a dearth of One Health or integrated training programs [1, 8]. One Health surveillance cannot be implemented and maintained effectively without qualified personnel. Inadequate quality health data, a low degree of usage of health information, and inadequate management of health information systems are obstacles to One Health surveillance in LMICs and prevent decision-makers at the community and district levels from using evidence-based planning and decision-making [9]. Clear communication with the public and other stakeholders is necessary for effective disease surveillance to raise awareness, stimulate reporting, and guarantee adherence to control measures. However, as an analysis for Kenya demonstrated, stakeholders’ unwillingness or incapacity to collaborate is a widespread problem, with opposition coming from slaughterhouse employees and animal owners in addition to underqualified medical professionals and ignorant patients [10]. One Health surveillance in LMICs faces difficulties in implementing and maintaining collaborative efforts for surveillance activities across stakeholders with different values, cultural upbringings, and interests, as well as difficulties in maintaining stakeholder participation in a participatory process to ensure the implementation of jointly developed solutions and assess their efficacy and impacts [11].

### Key Gaps in OHS Research in India

The literature review indicated that, limited formal coordination among veterinary, medical, and environmental professionals on the day-to-day prevention and detection of zoonotic diseases at district/sub-district levels [12]. Effective management of zoonotic diseases is disturbed by a poor understanding of the ecology of disease transmission [13]. A worldwide bibliometric analysis done by Miao et al. highlights that the number of publications on One Health has increased steadily from 2003 to 2021, with an annual growth rate of 20.2%; however, environmental health needs to be addressed in research on One Health [14]. OHS requires collaborative actions from all three sectors; hence, the role and responsibilities of each stakeholder should be mentioned in policy documents. However, the current surge in political and financial attention to One Health is weakened and splintered by power struggles between dominant human and animal health stakeholders, a lack of investment in collaboration-building skills, and a shift of operationalization in directions most aligned with stakeholders’ interests [15]. Many studies have focused on and implemented a One Health approach to prevent and control zoonotic disease outbreaks but neglected an early warning system. Health promotion and the identification of risk factors from the One Health perspective are often needed to address complex global issues like pandemics and climate change [16].

## Methods

### Study Type: Policy Content Analysis

This study is the first part of the earlier published study protocol by Yasobant S. et al., 2023 [17], which involves a secondary policy content analysis during 2023-24. In this study, the IDSP-IHIP as part of the human health surveillance and NADRS as part of the animal health surveillance policies were identified and included in the content extraction. Further, the selected zoonotic disease outbreak management guidelines were also included for the extraction. The content extraction aimed to fit the criteria for the matrix to evaluate multisectoral collaboration for One Health Surveillance Matrix (OHSM) developed by Bordier M et al. in 2019 [18]. The evaluation criteria for the matrix were based on the categories of collaborative strategy, modalities, coverage, resources, steering and coordinating mechanisms, scientific and technical support, training, information, monitoring and evaluation, engagement, surveillance design, sampling, laboratory activities, data and result sharing, data stock management, data analysis and interpretation, internal and external communication, and dissemination.

### IDSP: Human Health Surveillance System of India & NADRS: Animal Health Surveillance System of India (Governance Structure)

**Fig. 1:**
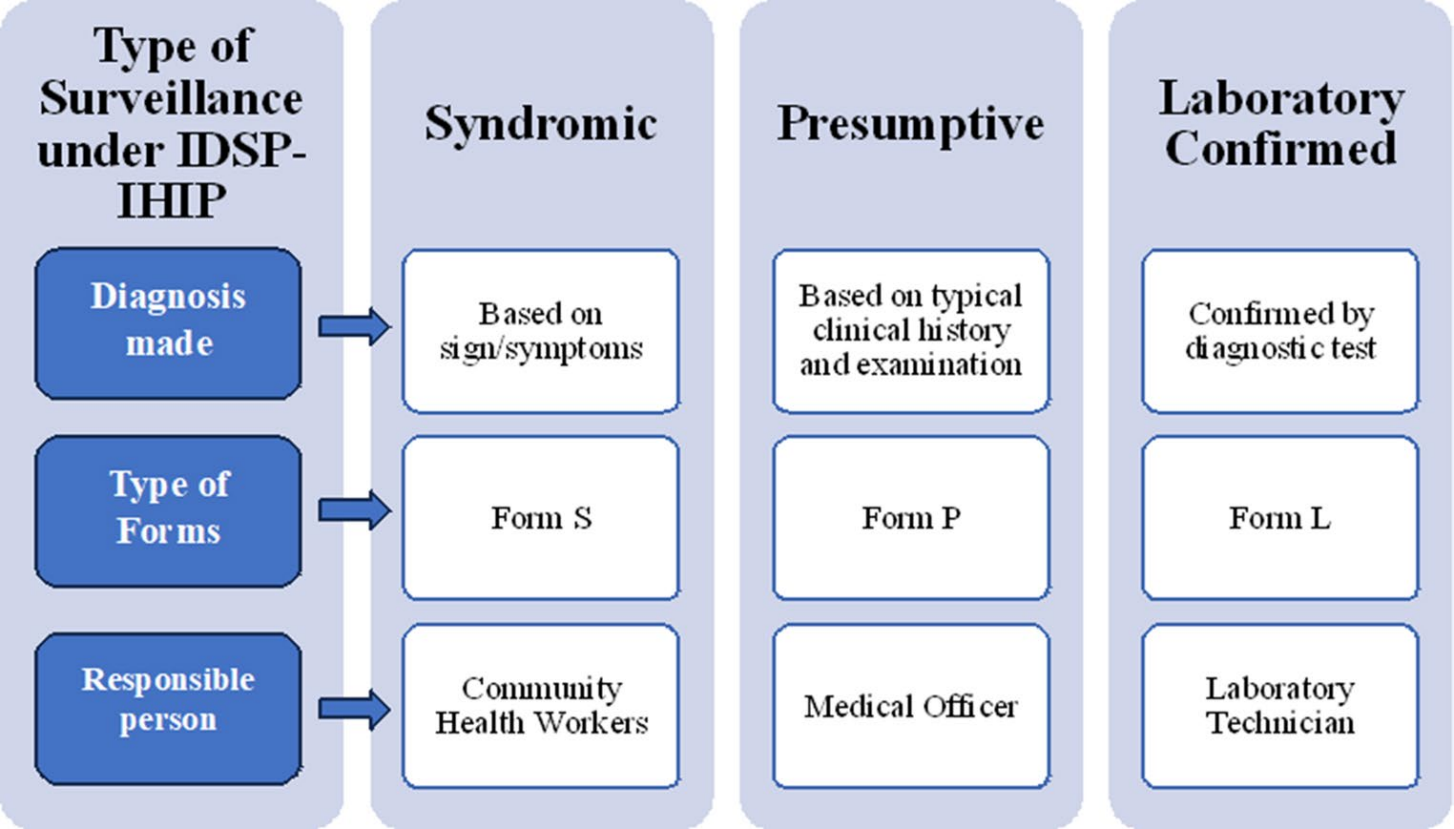
IDSP-IHIP surveillance forms

IDSP-IHIP is a state-based decentralized monitoring system for diseases susceptible to outbreaks. Its goal is to identify early warning signs so that effective public health measures may be taken to address health issues at the District, State, and National levels [19]. The portal of IDSP-IHIP was launched in 2018 with elements of web-based data collection, capturing disaggregate data of persons at all levels, electronically linked data of S (Syndromic Cases), P (Presumptive Cases), and L (Laboratory Confirmed Cases) forms, real-time data, and monitoring more than 33 health conditions (Fig. 1). NADRS is the animal disease reporting system with the primary objective of recording and monitoring livestock disease situations in India. Based on the modified NARDS 2.0 application, an android-based mobile application for capturing animal disease information, i.e., First Information Report (FIR), disease incidence (DI) cases, and vaccination coverage from the Block Veterinary Officers, with validation by District Veterinary Officers and the State Veterinary Officer has been developed.

In the 12th five-year plan, the Department of Health & Family Welfare, Ministry of Health and Family Welfare, Govt. of India implemented a new program for Strengthening Inter-sectoral Coordination for Prevention and Control of Zoonotic Diseases [20]. The primary objective of this program is to establish an intersectoral coordinating mechanism at the national, state, and district levels, to share relevant information with stakeholders, to develop a laboratory for diagnosis of zoonotic disease, and to develop capacity building and creating awareness among health and veterinary professionals about Zoonotic Diseases of Public Health Importance (ZPHI). 25 States have hired a veterinary consultant as part of the IDSP plan for intersectoral coordination.

### Study Process: Search of Policy Documents, Policy Content Extraction

The surveillance policies documents were retrieved by reviewer 1 (SY) and review 2 (RT) from the official websites of IDSP and NADRS. The documents obtained from the IHIP-IDSP includes manual and guidelines for different stakeholders i.e., Health Workers (HWs), Para-medical and Medical Officers, Veterinary Consultant, and Surveillance Officers, involved in disease surveillance activities. In addition, a manual for laboratory strengthening, guidelines for the collection, storage, and transportation of human clinical samples, a data management manual, and a data reporting and analysis manual are also included. Animal disease surveillance documents were downloaded from ICAR-NIVEDI, Department of Animal Husbandry, and Dairying, Department of Animal Husbandry.

### Study Data Extraction: Model Description and Extraction Details

The OHSM is a semi-quantitative tool used to analyze content extracted from policy documents [21]. The main objective of this tool is to evaluate the organization and functioning of current collaboration in a multisectoral surveillance system and to analyse its strengths and weaknesses. For evaluation of collaboration, OHSM has designed 22 organizational attributes, 9 functional attributes and three organizational indexes which aim to evaluate core characteristics of the organization on collaboration for governance and implementation of surveillance activities, core functions of collaboration, and organization of collaboration at a macro level, respectively. Then, 75 evaluation criteria that are scored using a four-tiered scoring grid (Grade 3,2,1 and 0) are used to determine to which degree these attributes and indexes are satisfied.

### Study Data Analysis: Brief about the Domains of the Analysis

The OHSM consists of four sheets in a spreadsheet. The first sheet has a scoring grid with four possible grades and a thorough scenario for each 75 criteria. The numerical results for each attribute and index are shown on the second sheet. It displays evaluation results for attributes and indexes using a formula:$\:\sum\:_{i}^{n}=0xi/3n$, where x i represents the grade, n represents the number of criteria, and 3 represents the highest score. Following scoring 23 organizational attributes, the OHSM generates three graphical representations of the evaluation results. In the first display, the results are presented as a pie graph, making it simple to identify the assessed attributes and their level of satisfaction. The index results are displayed in percentage terms of compliance with an ideal scenario in the second display. In the third display, the results of the nine functional attributes are shown on a five-tiered scale using a spider chart. A grade of A denotes a satisfaction level between 76 and 100%, while a grade of E denotes 0%. Its experience with more than 25 surveillance system assessments demonstrates the tool’s effectiveness [21]. These concepts are valuable and practical when evaluating surveillance systems using the OHSM. The final sheet includes graphical representations of the evaluation results along with all the formulas needed to score the attributes and indexes.

Both SY & RT independently extracted content from the selected policy documents and inserted relevant data in the OHSM matrix. Both have scored independently, using the grade scoring system for criteria in the OHSM, which ranged from grades 0 to 3 (Supplement). A third reviewer (WB/DS) resolved the conflict between these two reviewers and avoided subjective biases; hence, the final evaluation result is the consensus output between all reviewers.

## Results

### Evaluation of Result of Output 1: Organisational Attributes of Collaboration

**Fig. 2:**
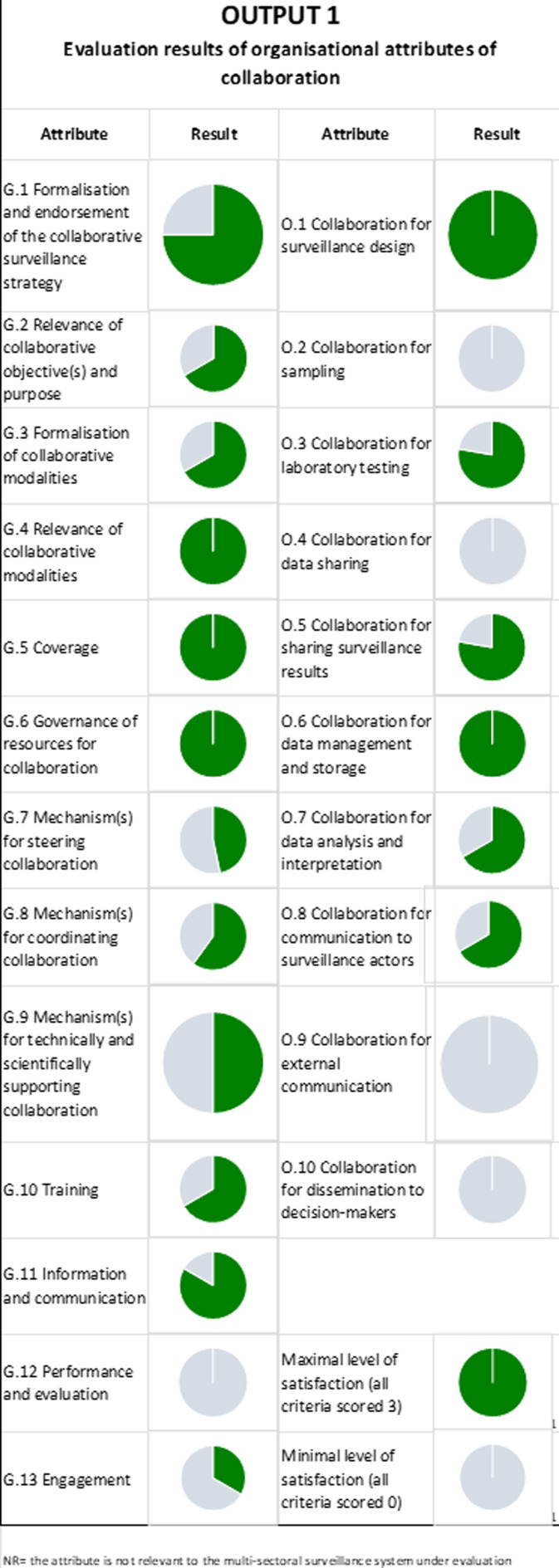
Evaluation results of organisational attributes of collaboration– IDSP

**Fig. 3:**
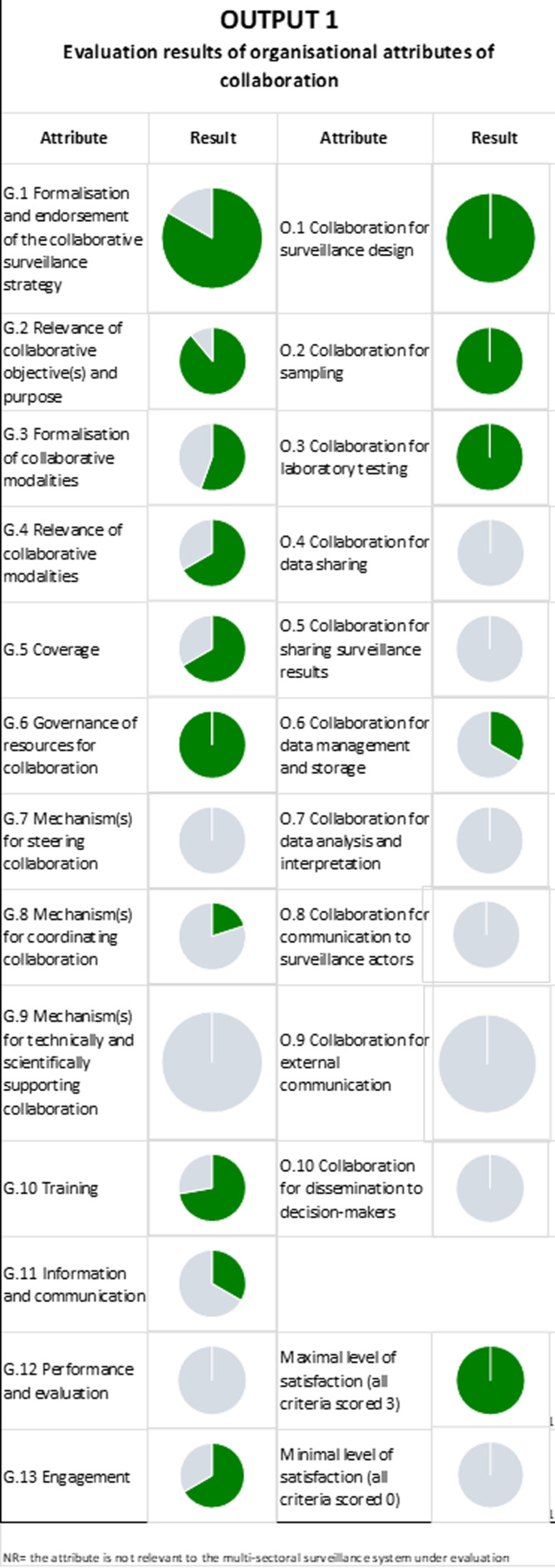
Evaluation results of organisational attributes of collaboration– NADRS

Output 1 shows the evaluation results of organizational collaboration attributes at the governance and organizational levels. According to these evaluation results, IDSP and NADRS need improvement in several areas. Weaknesses in the IDSP are found in data sharing, sampling, outreach to decision-makers, and external communication (Fig. 2). NADRS exhibits deficiencies in the following areas: data dissemination to decision-makers, management and storage of data analysis, interpretation of data, and external communication (Fig. 3). Animal husbandry and health departments should emphasize working together to address these deficiencies. Cooperative sample collection efforts, improved data-sharing accessibility through integrated systems, and cooperative report production might greatly boost collaboration between the two sectors.

### Evaluation of Result of Output 2: Organisational Indexes of Collaboration

**Fig. 4:**
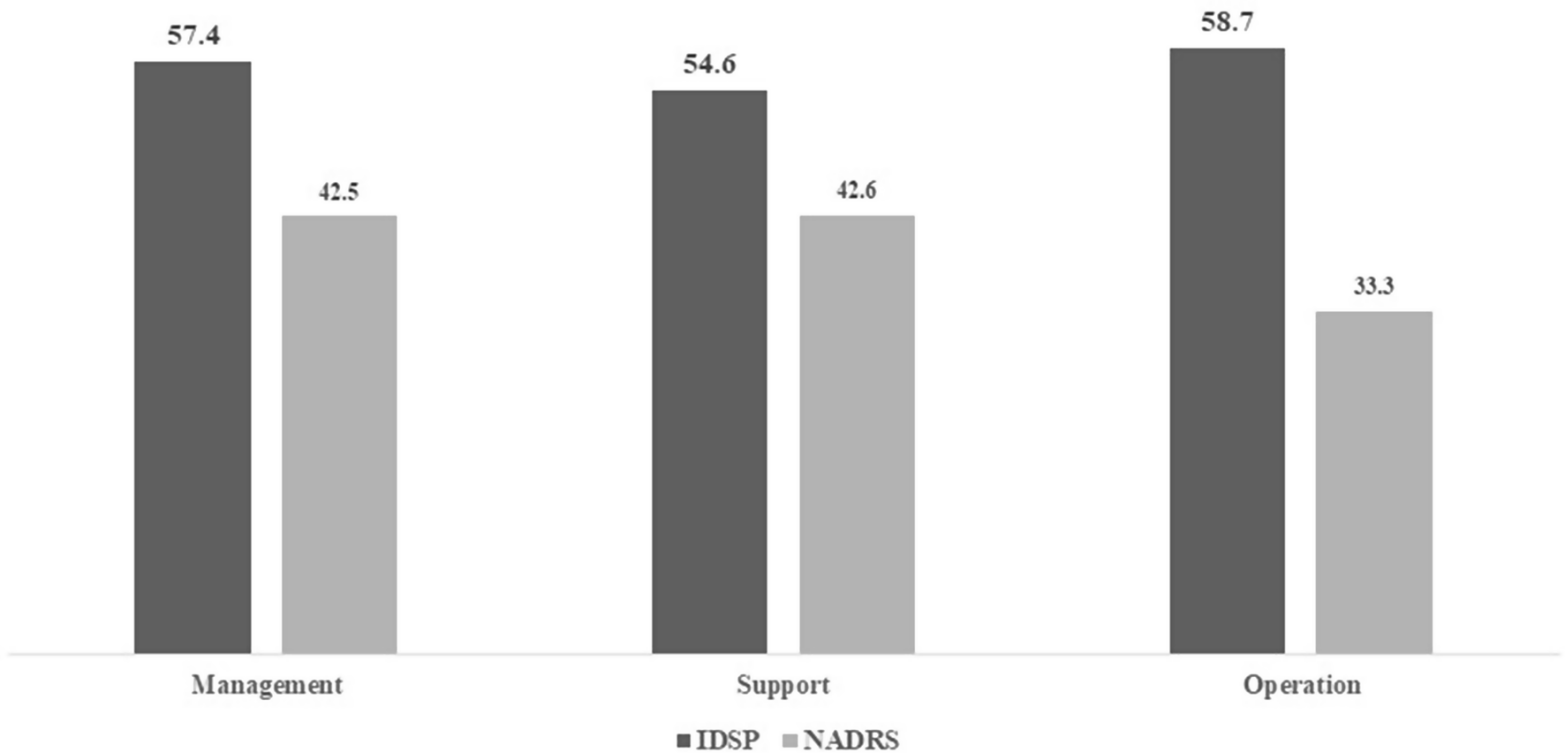
Evaluation results of organisational indexes of collaboration of IDSP & NADRS

The bar charts present the percentage of fulfilment of criteria contributing to the collaboration process. In IDSP, management represents 57.4%, and support and operation show 54.6% and 58.7%, respectively. While in NADRS, management represents 42.5%, and support and operation show 42.6 and 33.3%, respectively (Fig. 4).

### Evaluation of Result of Output 3: Functional Attributes

**Fig. 5:**
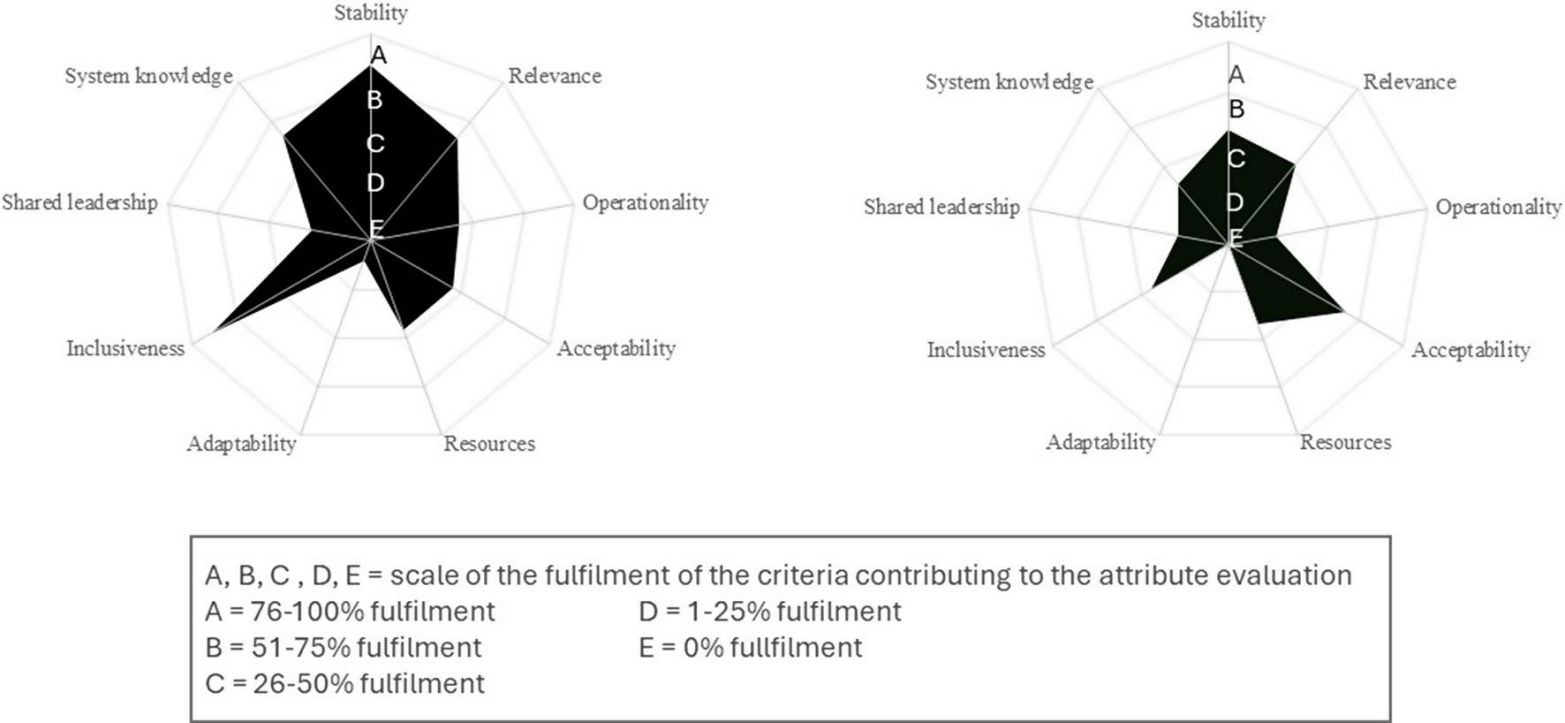
Evaluation results of functional attributes of collaboration (Left) IDSP, (Right) NADRS

The comparative assessment of the Integrated Disease Surveillance Program (IDSP) and the National Animal Disease Reporting System (NADRS) through the OHSM offers valuable insights into the collaborative multi-sectoral surveillance efforts in human and animal health. This result synthesizes the findings, highlights the strengths and weaknesses of each system, and provides recommendations for enhancing their effectiveness (Fig. 5).

#### Stability

IDSP emerges with a high stability level 79–100%, underscoring its robust and enduring collaboration. This stability is pivotal for sustained disease surveillance, especially in the context of zoonotic diseases with global implications. On the other hand, NADRS exhibits moderate stability 51–75%, indicating a reasonably firm foundation but with room for improvement.

#### Relevance

Both systems fall within the 26–50% range for relevance, suggesting the need for refinement in aligning collaborative strategies and activities with their overall objectives and contexts. Enhancing relevance is crucial for ensuring the impact of health interventions, and strategic adjustments in both IDSP and NADRS are recommended to achieve this alignment more effectively.

#### Operational

IDSP and NADRS present opportunities for increased operational efficiency 26–50% and 1–25%, respectively. The significant gap in operationality for NADRS calls for immediate attention to enhance its overall functionality.

#### Adaptability

The lack of adaptability in IDSP and NADRS 0% and absence signals a critical improvement area. In the face of evolving governance modalities, knowledge, and contextual changes, both systems need to develop mechanisms that enable flexibility and responsiveness.

#### Inclusiveness

IDSP exhibits high inclusiveness 76–100%, demonstrating effective involvement of relevant stakeholders. NADRS, while within a moderate range 26–50%, requires strengthening in role allocation based on mandates and competencies. Both systems can benefit from ensuring comprehensive representation to foster a collaborative approach to health monitoring.

#### Resource

Resource utilization falls within the 26–50% range for both IDSP and NADRS, indicating potential for optimization. Clear resource allocation, appropriateness, and availability mechanisms are recommended enhancements for both systems.

#### Shared Leadership

IDSP shows moderate success in shared leadership 26–50%, indicating room for improved collaboration among different stakeholders. NADRS, however, operates at a lower level 1–25%, highlighting a significant area for improvement. Strengthening governance mechanisms to create a trustworthy environment is vital for fostering mutual understanding and shared leadership in both systems.

#### System Knowledge

While IDSP exhibits a relatively high level of system knowledge 51–75%, NADRS falls within the 26–50% range, indicating a reasonable but improvable understanding. Enhancing institutional memory and communication systems is recommended for both systems to facilitate more effective data sharing at relevant levels.

### Case Study 1: Rabies Elimination in Gujarat

Rabies is a fatal disease affecting both humans and animals in India. The country aims to eliminate dog-mediated rabies by 2030 under the National Action Plan (NAPRE) [22], adopting a One Health approach that fosters collaboration between multiple agencies. The Rabies Free Cities strategic plan exemplifies this approach by facilitating coordination among the Health Department, Animal Husbandry Department, and municipal authorities. Key initiatives include mass dog vaccination and ensuring the availability of anti-rabies vaccines (ARV) and anti-rabies serums (ARS) at health centers for animal bite victims. Stakeholder roles and engagement are clearly defined, though the need for a more structured data-sharing mechanism remains. The program’s governance is rated highly due to efficient coordination between departments. For instance, the Health Department ensures continuous ARV and ARS availability, while the Animal Husbandry Department focuses on achieving a 70% dog vaccination rate in high-risk areas.

Intersectoral collaboration was assessed using the OHS matrix grading system. Collaboration and resource allocation received Grade 3, indicating that the objectives and responsibilities are well-documented and implemented effectively. However, role and responsibility assignment received Grade 2, as although most roles are outlined, some need more clarity and detail. Data sharing was rated Grade 1, reflecting significant gaps in resource mobilization and discrepancies between needs and provisions. In conclusion, while the program demonstrates strong collaboration in certain areas, its effectiveness is hindered by unclear role assignments and inadequate data-sharing mechanisms, which are critical for successful rabies control. Overall, the NAPRE action plan illustrates that the Eliminate program has satisfactory management and support, ensuring smooth coordination among various stakeholders; however, the lack of data-sharing mechanisms makes it challenging to generate relevant collaborative outputs. With regards to functional attributes, all attributes show the level of satisfaction for each core collaborative function except operationality due to the existence of a structured data-sharing mechanism and suggest having specific contraptions except periodic reports.

### Case Study 2: Avian Influenza

The contingency plan for avian influenza covers several key aspects related to managing human cases of avian influenza [23]. It emphasizes collaborative strategies, with multiple agencies such as the Department of Animal Husbandry and the Ministry of Health working together on surveillance design and containment efforts. It outlines the training provided to cullers and field workers and highlights sampling and laboratory activities, including guidelines for specimen collection and testing, which gives it Grade 3. Monitoring and evaluation are addressed through health checks for cullers and daily reporting by rapid response teams. Data and result sharing are included, particularly in relation to sharing findings with the National Influenza Pandemic Committee. Internal and external communication is mentioned, with the National Institute of Communicable Diseases (NICD) responsible for coordinating communication with domestic and international bodies like the World Health Organization.However, the document needs more specific references to formal steering and coordinating mechanisms and scientific and technical support beyond rapid response teams. It also does not elaborate on broader resource allocation or community engagement strategies, which gives it Grade 2. While surveillance is covered, detailed steps for data stock management, analysis, and interpretation are not provided, which led to Grade 2. Additionally, although public information is mentioned, structured dissemination of scientific findings beyond immediate response actions is not addressed; thus, it received Grade 1.

Overall, the avian influenza contingency plan shows satisfactory management collaboration; however, support and operation collaboration could be improved due to the above-mentioned discrepancies. Further, regarding the functional attributes, it shows that the surveillance design, training, and internal and external communication have better collaborative functions in the context of acceptability, stability, inclusiveness, operationality, and relevance than data sharing, analysis, dissemination of information, which shows need for improvement in resource allocation, shared leadership and system knowledge.

## Conclusion & Recommendations

In conclusion, this OHS assessment on the policy content analysis of the IDSP & NADRS indicated that multiple scope for improving collaborations is ultimately crucial for improving disease surveillance and response efforts. Both systems share common weaknesses, particularly in areas such as data sharing, dissemination, and external communication, as profoundly documented in this analysis. For IDSP, the focus should be on enhancing engagement, monitoring and evaluation, and sampling processes, with a special emphasis on the joint collaborative effort with the animal health sectors. On the other hand, NADRS needs improvement in coordinating mechanisms, data analysis and interpretation, internal communication, result sharing, scientific and technical support, and steering mechanisms, with a special emphasis on the close coordination with the health sectors. Nevertheless, these weaknesses can be addressed by implementing a joint real-time online platform for zoonotic disease reporting, allowing relevant stakeholders to monitor data and collaborate more effectively. Additionally, a structured mechanism should be established for disseminating findings and results from the joint surveillance data, including predicting early warning and response systems. The findings from this study will be helpful to both human and animal health policymakers in drafting robust collaboration strategies for developing a One Health surveillance system in India.

By focusing on the identified weak points and implementing targeted strategies for joint action and communication, both programs can strengthen their effectiveness in detecting, managing, and mitigating disease outbreaks. IDSP demonstrates higher stability, inclusiveness, and system knowledge, positioning it as a more robust collaborative system. NADRS exhibits notable areas for improvement, particularly in operationality, adaptability, shared leadership, and relevance. Recommendations include refining collaborative strategies, streamlining operational governance, enhancing adaptability, strengthening inclusiveness, and optimizing resource allocation mechanisms. By addressing these areas, both IDSP and NADRS can further strengthen their collaborative efforts, ultimately contributing to more impactful and evidence-based health interventions in the surveillance of zoonotic diseases and animal health reporting. This comparative analysis serves as a roadmap for strategic enhancements in both systems, fostering a more resilient and effective multi-sectoral surveillance landscape.

## Electronic Supplementary Material

Below is the link to the electronic supplementary material.

Supplementary Material 1

## Data Availability

No datasets were generated or analysed during the current study.

## Abbreviations

ARV: Anti-Rabies Vaccines
ARS: Anti-Rabies Serums
CDC: Centre for disease control
DI: Disease incidence
FIR: First information reporting
HWs: Health workers
ICAR-NIVEDI: Indian council of agricultural research - national institute of veterinary epidemiology and disease informatics
IDSP: Integrated disease surveillance program
IHIP: Integrated health information program
JE: Japanese encephalitis
KFD: Kyasanur forest disease
LMIC: Low- and middle-income country
NADRS: National animal disease reporting system
NAPRE: National action plan for the elimination of dog-mediated Rabies
NICD: National institute of communicable diseases
OHS: One health surveillance
OHSM: One health surveillance matrix
ZPHI: Zoonotic diseases of public health importance
